# Abnormal global alternative RNA splicing in COVID-19 patients

**DOI:** 10.1371/journal.pgen.1010137

**Published:** 2022-04-14

**Authors:** Changli Wang, Lijun Chen, Yaobin Chen, Wenwen Jia, Xunhui Cai, Yufeng Liu, Fenghu Ji, Peng Xiong, Anyi Liang, Ren Liu, Yuanlin Guan, Zhongyi Cheng, Yejing Weng, Weixin Wang, Yaqi Duan, Dong Kuang, Sanpeng Xu, Hanghang Cai, Qin Xia, Dehua Yang, Ming-Wei Wang, Xiangping Yang, Jianjun Zhang, Chao Cheng, Liang Liu, Zhongmin Liu, Ren Liang, Guopin Wang, Zhendong Li, Han Xia, Tian Xia

**Affiliations:** 1 Department of Pathology, School of Basic Medicine, Tongji Medical College, Huazhong University of Science and Technology, Wuhan, China; 2 Institute of Artificial Intelligence and Automation, Huazhong University of Science and Technology, Wuhan, China; 3 Institute for Regenerative Medicine, Shanghai East Hospital, School of Life Sciences and Technology, Tongji University, Shanghai, China; 4 Department of Research and Development, Hugobiotech Co. Ltd., Beijing, China; 5 Jingjie PTM BioLab (Hangzhou) Co. Ltd., Hangzhou, China; 6 Department of Pathology, Union Hospital, Tongji Medical College, Huazhong University of Science and Technology, Wuhan, China; 7 The National Center for Drug Screening, Shanghai Institute of Materia Medica, Chinese Academy of Sciences, Shanghai, China; 8 Department of Thoracic/Head and Neck Medical Oncology, The University of Texas MD Anderson Cancer Center, Houston, Texas, United States of America; 9 Department of Genomic Medicine, The University of Texas MD Anderson Cancer Center, Houston, Texas, United States of America; 10 Department of Medicine, Baylor College of Medicine, Houston, Texas, United States of America; 11 Department of Forensic Medicine, Tongji Medical College, Huazhong University of Science and Technology, Wuhan, China; 12 Elongevity INC, Wuhan, China; 13 School of Automation Science and Engineering, Faculty of Electronic and Information Engineering, Xi’an Jiaotong University, Xi’an, China; University of Miami, Miller School of Medicine, UNITED STATES

## Abstract

Viral infections can alter host transcriptomes by manipulating host splicing machinery. Despite intensive transcriptomic studies on SARS-CoV-2, a systematic analysis of alternative splicing (AS) in severe COVID-19 patients remains largely elusive. Here we integrated proteomic and transcriptomic sequencing data to study AS changes in COVID-19 patients. We discovered that RNA splicing is among the major down-regulated proteomic signatures in COVID-19 patients. The transcriptome analysis showed that SARS-CoV-2 infection induces widespread dysregulation of transcript usage and expression, affecting blood coagulation, neutrophil activation, and cytokine production. Notably, *CD74* and *LRRFIP1* had increased skipping of an exon in COVID-19 patients that disrupts a functional domain, which correlated with reduced antiviral immunity. Furthermore, the dysregulation of transcripts was strongly correlated with clinical severity of COVID-19, and splice-variants may contribute to unexpected therapeutic activity. In summary, our data highlight that a better understanding of the AS landscape may aid in COVID-19 diagnosis and therapy.

## Author summary

Despite intensive studies on the transcriptional signatures of COVID-19 patients, how SARS-CoV-2 affects AS landscape and the contribution of AS to the pathogenesis of COVID-19 remain largely elusive. By profiling the lung transcriptome and lung proteome of nine patients who died of COVID-19 during the first wave of the pandemic in Wuhan, China, we obtained molecular insights into the AS of cellular transcripts upon SARS-CoV-2 infection. Interestingly, SARS-CoV-2 proteins directly engage host spliceosome to dysregulate essential steps of mature mRNA production and result in widespread dysregulation of cellular function. Taken together, our findings shed light on COVID-19 molecular mechanism and offer potential therapeutic targets for severe COVID-19 disease.

## Introduction

The current COVID-19 pandemic caused by SARS-CoV-2 poses significant challenges for not only global public health but also life normalcy [1]. While significant progresses have been made for the management of the disease, treatments are mainly supportive and symptomatic care [2,3]. Understanding the interaction between host and SARS-CoV-2 will provide new insights on COVID-19 pathogenesis and the development of effective antiviral therapies [4].

Alternative splicing (AS) is a fundamental mechanism for the regulation of proteome diversity through the splicing of a single RNA to generate alternative mRNAs encoding structurally and functionally distinct protein isoforms [5]. Multiple viruses hijack this pathway to favor their replication and evade host’s antiviral responses. Ddx58 protein of SARS-CoV-2 virus [6], NS5 protein of dengue virus [7], NS1 protein of influenza virus [8], and Vpr protein of HIV-1 [9] interact with the cellular spliceosome complex and inhibit the splicing reaction. On the other hand, viral infection can induce numerous AS of host RNAs that translate into altered proteins, which are critical for cell cycle, DNA synthesis, stress response nuclear transport, and immune responses [10–13]. For example, a virus-induced alternatively spliced isoform of *TBK1* disrupts the interaction between RIG-I and MAVS and inhibits anti-viral IFN-beta signaling [14]. CD45, a critical molecule for T cell activation and function, has also been shown to express the smallest isoform CD45RO after HIV infection [15,16].

To date, high-throughput sequencing data, especially transcriptomic and proteomic data, has been used to identify the genome-wide AS events. For transcriptomic data, a number of computational approaches have been developed to identify and quantify differentially spliced genes, such as LeafCutter [17], rMATS [18], and DEXSeq [19]. For proteomic data, approaches searched proteomic sequencing data against databases containing splice variant sequences and then confirmed the translation of a spliced sequence by detecting a peptide unique to that form [20]. In addition, the integration of transcriptomic and proteomic data has become a new way to identify the AS [21]. Recently, several studies have profiled the transcriptomes of cells, tissues and fluids from COVID-19 patients, which revealed the dysregulated type I and III interferon pathways, hyper-inflammatory responses and activation of humoral immunity [22–25]. Notably, NSP16 protein of SARS-CoV-2 was discovered to bind to the mRNA recognition domains of the U1 and U2 splicing RNAs and act to disrupt host global mRNA splicing and suppress host defenses [26]. Despite SARS-CoV-2 protein is reported to interact with cellular spliceosomal components, the global alteration of host gene splicing and the contribution of AS to the pathogenesis of COVID-19 remain largely elusive. Characterization of the splicing landscape upon SARS-CoV-2 infection in host cells may provide unique and novel insights on how SARS-CoV-2 regulates and hijacks the immune system for their evasion.

In this study, we integrated multiple-omics datasets to analyze the dysregulation of host splicing machinery and the alteration of transcript isoforms in COVID-19 patients. We further explored the impact of the altered AS landscape on clinical outcomes, and identified potential disruptions of drug-binding sites of target proteins in COVID-19 patients. Our study provides additional insights into the complexity of the splicing landscape upon SARS-CoV-2 infection, which could aid in COVID-19 diagnosis and therapy.

## Results

### COVID-19 patients had perturbation of host splicing machinery

**Fig 1:**
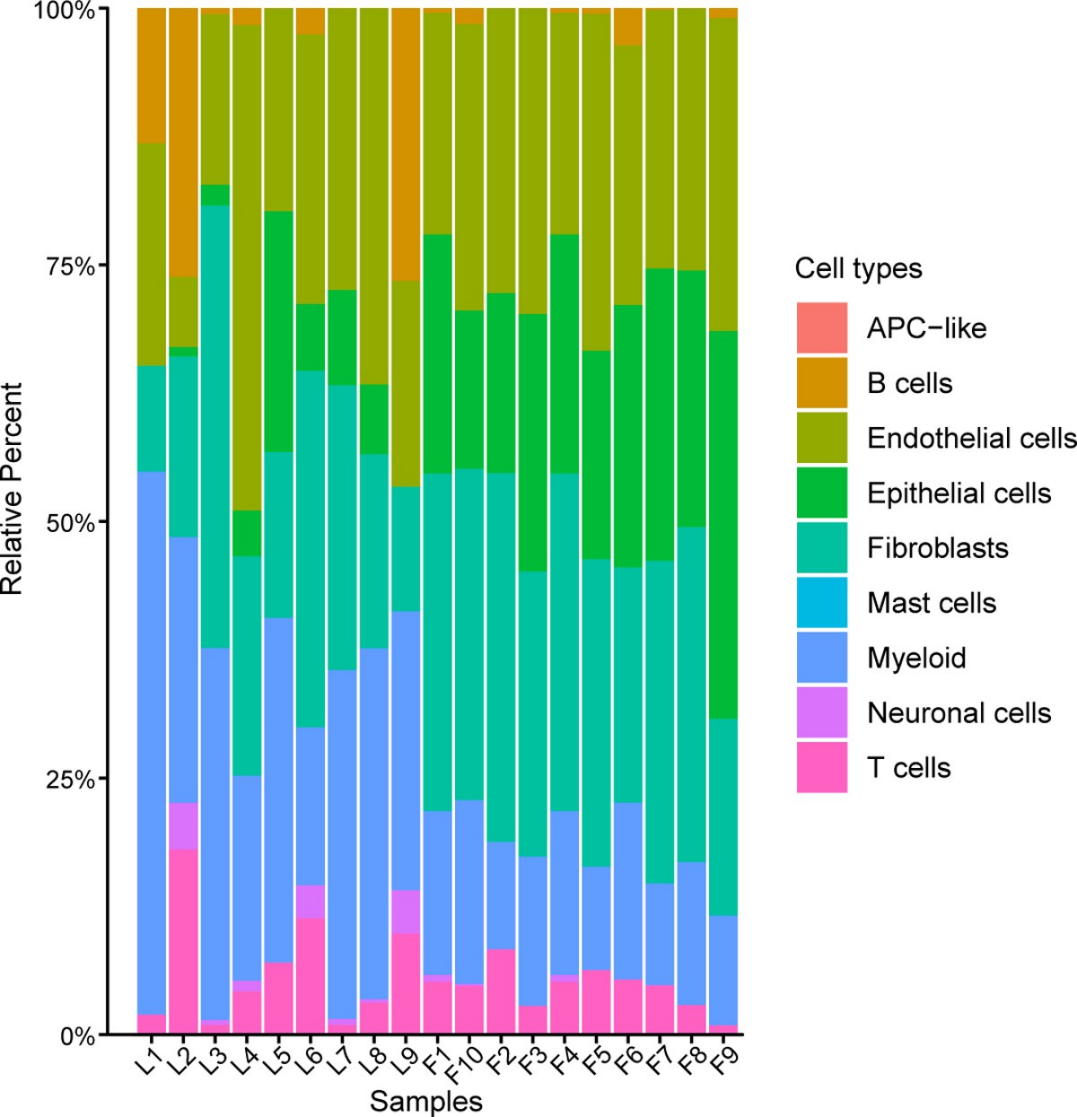
The proportion of 9 cell subpopulations in 9 COVID-19 and 10 control samples. The colors indicate cell type information. L1-L9, COVID-19 patients. F1-F10, control samples.

We obtained post-mortem lung samples from nine patients who died of COVID-19 in the early outbreak in Wuhan, China. Of these 9 patients, 5 were male, and the mean age was 68 years. The patients were symptomatic for an average of ~26 days prior to death. Given that this was during the initial outbreak, there was not a standard approach to treating patients, and they received a variety of antimicrobial and immunomodulatory medications. In our previous publication, we report the detailed clinical findings and histological evidence of these fatal COVID-19 lung samples [27,28]. All patients had histological evidence of diffuse alveolar damage, with widespread hyaline membrane formation, evidence of fibrosis, and varying degrees of inflammatory infiltrate. Immunohistochemical staining showed that these patients were noted to have a small number of CD4^+^ T cell, CD8^+^ T cell, CD20^+^ B cell and a significant number of CD68^+^ macrophages present in the lung tissue [27]. To estimate the cell type composition, we used the CIBERSORTx algorithm [29] to calculate the percentage of cell types in 9 COVID-19 and 10 control samples (see Materials and Methods for details). Consistent with our immunohistochemical staining observations, all COVID-19 patients had varying degrees of inflammatory infiltrate, with a large proportion of myeloid cells and a low proportion of T cells and B cells (Fig 1). Notably, 8 patients were noted to have a minor proportion of epithelial cells in the lung tissue (Fig 1). These observations are consistent with the distribution of major cell types observed upon a single-cell RNA sequencing (scRNA-seq) cohort which contained 116,000 cells from the lung autopsy samples of 19 individuals who died of COVID-19 and 7 control individuals [30].

**Fig 2:**
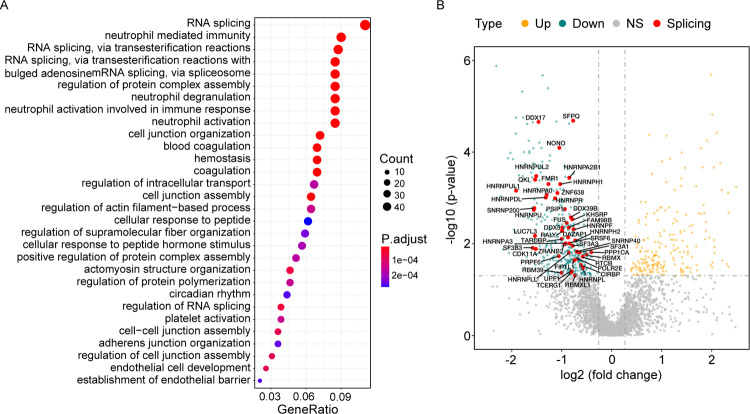
Proteomic profiling reveals dysregulated proteins in the lungs of COVID-19 patients. (A) Dotplot visualization of the top 30 enriched GO terms of downregulated proteins. Fisher’s exact test using enrichGO function in R package clusterProfiler. (B) A volcano plot (p value vs fold change) comparing gene expression in cases versus controls is shown at protein level. Up- and downregulated proteins are highlighted in orange and green, respectively and RNA splicing related proteins that are differentially expressed are named. The horizontal dashed line represents an adjusted p value of 0.05 (Wald test in DESeq2, multiple test correction by Benjamini-Hochberg (BH)), and the vertical dashed lines represent log2FC of -0.26 and 0.26.

To investigate the effect of SARS-CoV-2 infection on protein expression, we performed proteomic analysis using LC-MS/MS on the 9 postmortem lung samples (cases); as controls, archived histologically normal lung tissue obtained from 10 SARS-CoV-2 uninfected individuals that underwent biopsy or surgical resection as part of routine clinical care for lung cancer. In total, we detected 4,689 proteins by mass spectrometry. Out of these proteins, we identified 235 upregulated and 402 downregulated proteins in the COVID-19 patients with respect to the controls (S1 Table). By GO enrichment analysis, we identified five splicing-related GO terms as the top enriched terms in the significantly downregulated proteins (Fig 2A). Specifically, two small nuclear ribonucleoprotein U5 subunits (SNRNP40 and SNRNP200) and multiple splicing factors (e.g., HNRNP A0, A2B1, A3, DL, F, H1, H2, L, LL, R, U, UL1, UL2 and SRSF6) were significantly under-expressed in the COVID-19 patients (Fig 2B). In addition, four neutrophil related terms were also found to be enriched in both up- [28] and down-regulated proteins (Fig 2A), suggesting serious dysregulation of neutrophil process in severe COVID-19 patients. Some of the processes identified, such as ‘blood coagulation’, ‘hemostasis’, and ‘coagulation’ (Fig 2A), which contain proteins relevant to hemorrhagic lesions, are not unexpected in severe COVID-19 patients.

Next, we investigated the virus-host protein-protein interaction (PPI) network using data from the VirHostNet database [31] and extracted viral proteins that directly interact with human splicing factors and spliceosomal components. Interestingly, among these known virus-host PPIs, 13 splicing factors were downregulated in the COVID-19 patients, including three members of RNA helicase, DDX5, DDX17 and DDX1 (S1 Fig). Together, these results suggest that host splicing machinery is profoundly dysregulated owing to SARS-CoV-2 infection, which could affect host immune response by altering AS of cellular genes.

### SARS-CoV-2 infection induced widespread transcriptional dysfunction

**Fig 3:**
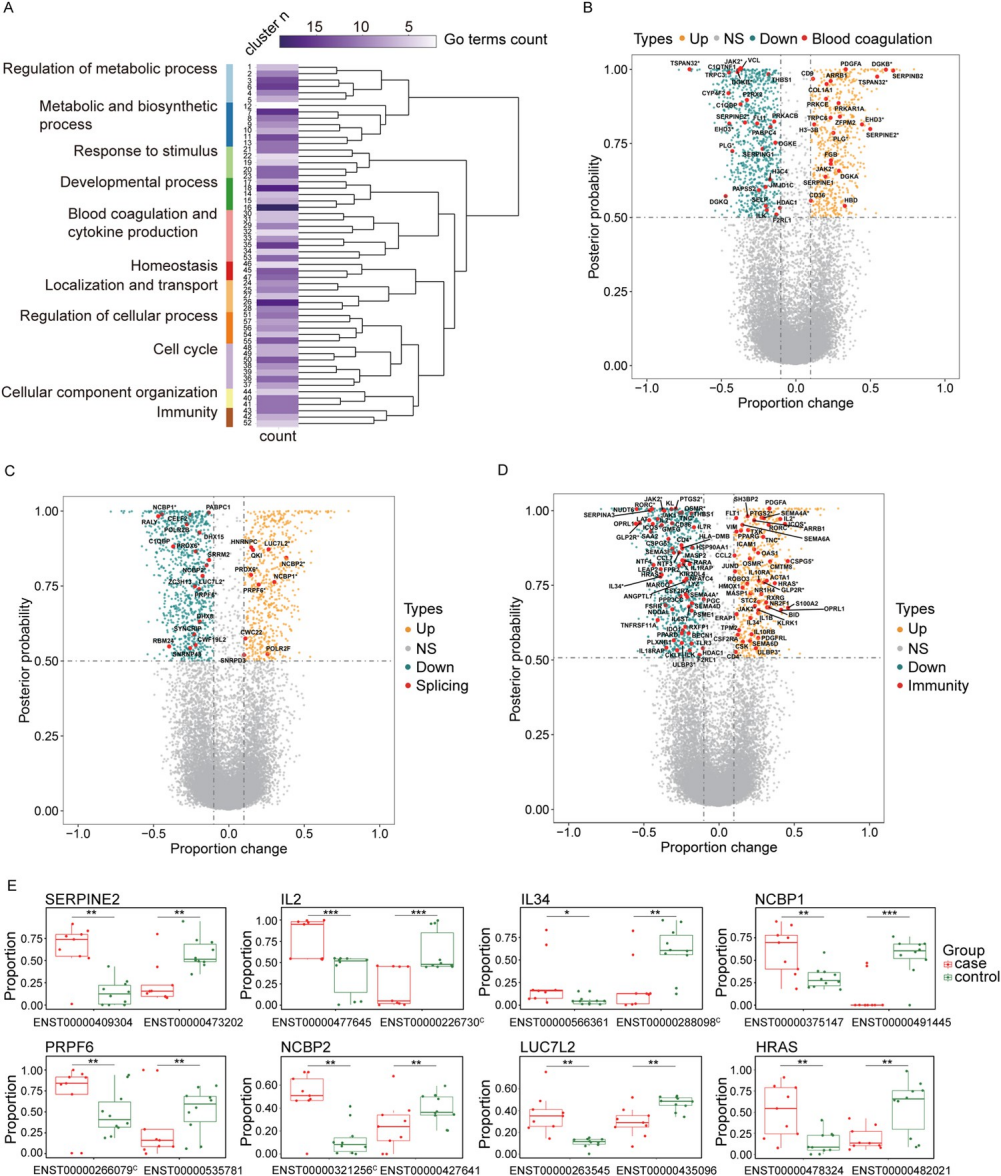
Transcriptional profiling reveals widespread dysfunction of cellular gene expression in the lungs of fatal COVID-19 patients. (A) Functional analysis of 1383 genes with significant DTU. The clustering heatmap plot of functional sets of GO terms was obtained using ViSEAGO showing the major biological processes, cluster number and number of GO terms in each cluster. Based on BMA semantic similarity distance and Ward’s clustering criterion. (B-D) A volcano plot (posterior probability vs. proportion change) comparing gene transcript usage in cases versus controls is shown at mRNA level. DTUs that were related to blood coagulation (B), RNA splicing (C) and immunity (D) are named. The horizontal dashed line represents posterior probability of 0.5, and the vertical dashed lines represent proportion change of -0.1 and 0.1. * represents isoform switch. (E) Isoform switch of *SERPINE2*, *IL2*, *IL34*, *NCBP1*, *PRPF6*, *NCBP2*, *LUC7L2* and *HRAS*. ^C^ represents canonical isoform. * posterior probability > 0.5; ** posterior probability > 0.7; *** posterior probability > 0.9.

To investigate whether there was a systematic dysregulation of host gene expression at the transcriptome level in fatal COVID-19 patients, we performed bulk RNA sequencing to evaluate the alteration of transcript usage in 9 postmortem lung samples versus 10 controls. We identified 1,383 genes whose major transcript (the most highly expressed transcript) displayed differential transcript usage (DTU) changes between the COVID-19 patients and the controls. GO enrichment analysis identified a total of 540 enriched terms among the 1,383 genes. These terms were grouped into 57 clusters that were further categorized into 11 major processes: blood coagulation and cytokine production, immunity, regulation of metabolic process, metabolic and biosynthetic process, response to stimulis, developmental process, homeostasis, localization and transport, regulation of cellular process, cell cycle and cellular component organization (Fig 3A). Out of these clusters, two (C33 and C34) were associated with ‘cytokine and chemokine production’ and ‘blood coagulation’, respectively (S2 Table).

DTU genes in these blood coagulation pathways included *SERPINB2*, *SERPINE2*, *SERPING1*, *DGKE*, *DGKQ*, *DGKB* and *DGKA* (Figs 3B and S2), which were involved in the generation of serine protease inhibitor (SERPIN) and diacylglycerol kinase (DGK). Of note, we observed numerous DTU genes involved in RNA splicing, with 10 genes (e.g. *HNRNPC*, *SNRPD3*, *QKI*, and *LUC7L2*) increased major transcript usage and 18 genes (e.g. *SNRNP48*, *NCBP1*, *CELF2*, *RALY*, and *PABPC1*) decreased major transcript usage in the COVID-19 patients (Figs 3C and S3). Although patients with severe COVID-19 have been found to have a cytokine storm in peripheral blood and bronchoalveolar lavage fluid [32,33], in lung parenchyma we only identified a small number of cytokine genes with differently major transcript usage, including *IL1B*, *IL2*, *IL6ST*, *IL34*, *CCL2*, *CCL7*, *CMTM8*, *CSPG5*, *PDGFA*, and *PDGFRL* (Figs 3D and S4). Furthermore, we observed that the major transcript of multiple genes switched to a different transcript (isoform switching) between COVID-19 patients and controls, such as *IL2*, *IL34*, *SERPINE2*, *NCBP1*, *HRAS*, *PRPF6*, *NCBP2*, and *LUC7L2* (Fig 3E). The canonical transcript usage of *IL2* was significantly decreased and the ENST00000477645 (noncoding protein) transcript usage was significantly increased in COVID-19 cases. Notably, *IL-2* plays a key role in the proliferation of T cells and in the generation of effector and memory T cells [34], suggesting that the transcript switch of *IL2* may affect the activation of T cells in severe COVID-19 patients.

Next, we expanded our analyses to the transcript isoform level and observed 3,937 differential transcript expression (DTE), with 1,890 up-regulated and 2,047 down-regulated transcripts in the fatal cases when compared to healthy controls. GO enrichment analysis on these up-regulated DTE genes identified four neutrophil-related GO terms as top enriched terms (S5A Fig), which is consistent with our previous observation of increased neutrophils in the lungs of fatal COVID-19 patients [28]. Notably, numerous genes with up-regulated transcript were related to oxidative stress response, which is consistent with clinical symptom of severe breathing difficulties in the fatal COVID-19 patients (S5A Fig). Additionally, ‘RNA catabolic process’, ‘mRNA catabolic process’, ‘regulation of DNA metabolic process’ and ‘RNA localization’ were significantly up-regulated in the COVID-19 patients, suggesting that SARS-CoV-2 reshapes central cellular pathways such as translation, carbon metabolism (S5A Fig). Surprisingly, the top GO terms that were down-regulated in these lung tissue samples represent neurobiological processes: axonogenesis, regulation of neuron projection development and axon guidance, suggesting that dysregulation of bidirectional interactions between lung and brain in fatal COVID-19 patients (S5B Fig).

### AS events identified in lung tissues of COVID-19 patients

**Fig 4:**
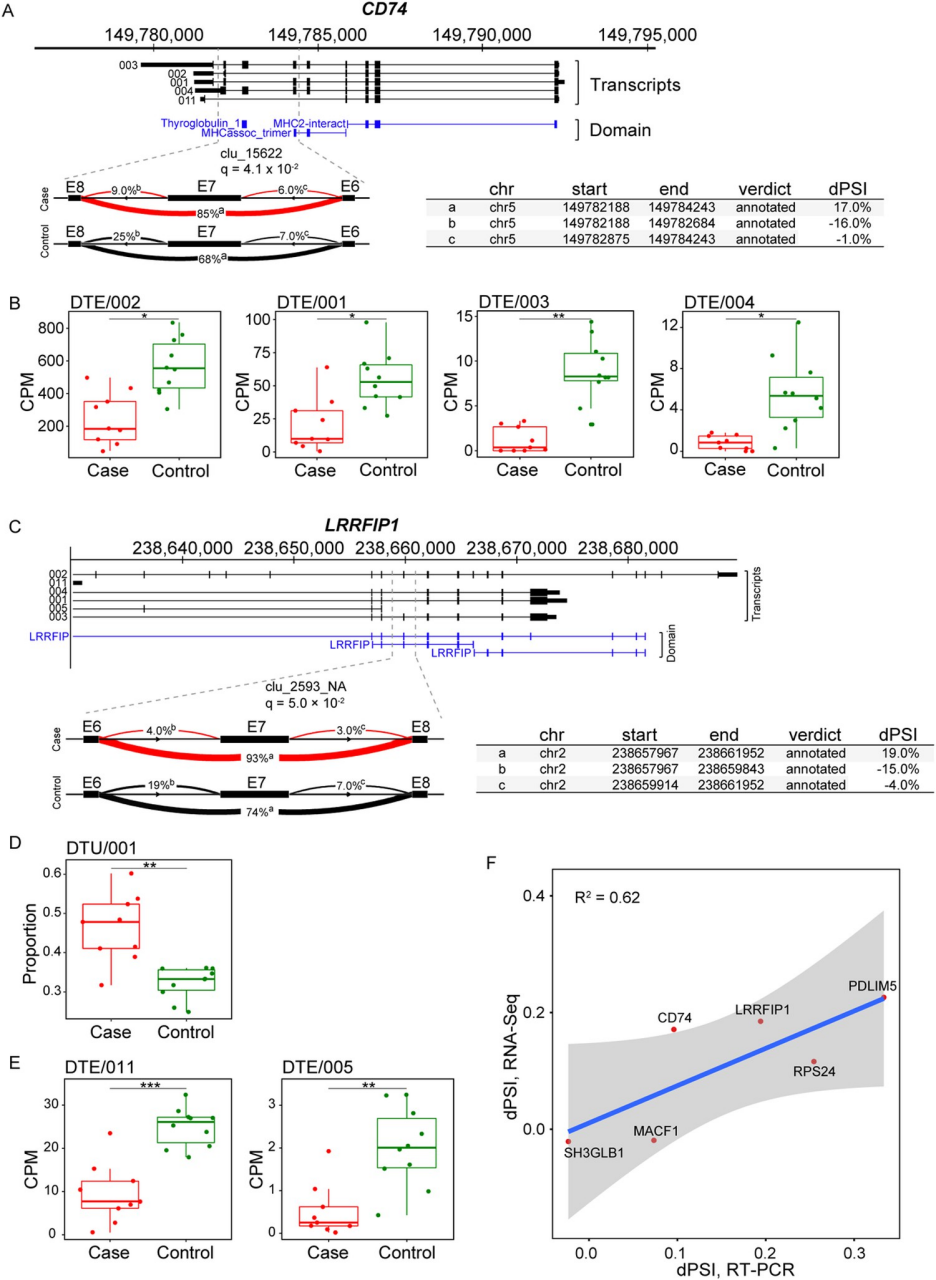
Aberrant local splicing and transcript isoform usage in COVID-19. (A) A significant differential splicing intron cluster in *CD74* (clu_15622; chr5: 149,782,188–149,784,243) showing increased exon 7 (E7) skipping in COVID-19. Increased intron usage in COVID-19 cases compared to control is highlighted in red. Protein domains are annotated as thyroglobulin_1, thyroglobulin type 1 repeats; MHCassoc_trimer, Class II MHC-associated invariant chain trimerisation domain; MHC2-interact, MHC2 interacting. Visualization of splicing events in cluster clu_15622 with changes in PSI (dPSI) for COVID-19 group comparisons. FDR-corrected P values (q) are indicated for each comparison. Covariate-adjusted average PSI levels in cases (red) versus controls (black) are indicated at each intron. (B) Bar plots for changes in gene expression for CD74-002, CD74-001, CD74-003 and CD74-004. CD74-003 is the canonical sequence. * posterior probability > 0.5; ** posterior probability > 0.7; *** posterior probability > 0.9. (C) Whole-gene view of *LRRFIP1* highlighting (dashed lines) the intron cluster with significant differential splicing in COVID-19 (clu_2593_NA; chr2: 238,657,967–238,661,952). LRRFIP, Leucine-rich repeat flightless-interacting protein domain. (D and E) Bar plots for changes in transcript usage and gene expression for *LRRFIP1* transcripts. * posterior probability > 0.5; ** posterior probability > 0.7; *** posterior probability > 0.9. (F) Scatter plots comparing the average percent spliced-in (PSI) of exon skipping events called by LeafCutter from RNA-seq data to semi-quantitative PCR. A total of 6 genes were tested in 9 COVID-19 cases and 10 controls. Gene names are indicated at each point. Regression lines with 95% confidence intervals are shown in blue and grey, respectively and the corresponding R^2^ values are shown at the top-left of the plot.

Given the profound degree of splicing related protein changes observed in our proteomic data, we sought to determine how many cellular genes with AS events within these severe COVID-19 patients. Using the LeafCutter [17] analysis, we identified a total of 402 intron clusters (corresponding to 366 genes) that displayed altered splicing in the COVID-19 patients (S3 Table). The most commonly observed local splicing change was exon skipping (ES, 41.8%), followed by complex event (36.6%), intron retention (10.3%), alternative 3’ splice site (5.9%) and alternative 5’ splice site (5.4%) (S3 Table). Additionally, many AS events show predictable functional consequences on protein isoforms. For example, isoform 001–004 of *CD74* are down-regulated in the COVID-19 patients (Fig 4B). We observed that an ES event in E7 (q = 4.1 × 10^−2^, dPSI = 17.0%) disrupts a thyroglobulin domain of *CD74* (Fig 4A), changes that are predicted to encode a truncated protein deficient in its ability to induce antiviral activity. As another example, we observed significant AS, DTU, and DTE changes in *LRRFIP1* transcripts (Fig 4C and 4E). The cytosolic nucleic acids sensor LRRFIP1 mediates the production of type I interferon, which plays an important role in exacerbating TNF- and IL-1-driven inflammation in the progression to severe COVID-19 [35]. An ES event in E7 (q = 5.0 × 10^−2^, dPSI = 19.0%) disrupts a LRRFIP domain in *LRRFIP1*, changes that are predicted to have major effects on its function. In addition, we deployed validation experiments for local splicing changes of 6 genes (*CD74*, *LRRFIP1*, *SH3GLB1*, *MACF1*, *RPS24* and *PDLIM5*) between 9 COVID-19 cases and 10 controls by semiquantitative reverse transcription (RT)-PCR. The result showed percent spliced-in (PSI) changes consistent with those reported by LeafCutter (Figs 4F and S6). To validate DTE results, we performed similar semiquantitative RT-PCR on 4 selected transcripts (CD74-001, CD74-002, LRRFIP1-011, and LRRFIP1-005) and found concordance in transcript expression changing pattern (except for CD74-002) between RNA-seq data analysis and validation experiments (S7A and S7B Fig). Overall, this examination of local AS events in fatal COVID-19 patients, coupled with the analysis of isoform-level regulation, emphasizes the need to understand the critical impact of transcript isoform regulation on gene function.

Next, we created an independent gene co-splicing network in the cases and controls respectively through the MONET K1 method, which detected COVID-19 associated modules by kernel clustering with diffusion state distance as metric [36]. Although calculated separately, the case and control networks have similar genes and modules, and generally reflected equivalent biological processes. However, the co-splicing connections showed a great divergent network between the case and control groups. For example, the neutrophil activation process enriched in both case and control networks and exhibited a completely different structure and connection (S8A Fig), suggesting that there are significant phenotypical alterations of neutrophil from COVID-19 patients compared to healthy controls. Notably, DDX3X, the hub of neutrophil activation network, encodes a DEAD-box RNA helicase with putative roles in RNA metabolism, cell cycle progression, apoptosis, and viral immunity [37]. In addition, the 18th module shows greater enrichment for macrophage activation in cases and the 48th module is enriched for genes harboring B cell homeostasis in controls (S8B and S8C Fig), which could be used to distinguish fatal COVID-19 patient and healthy control.

### The degree of splicing deregulation is strongly correlated with severity of COVID-19 patients

**Fig 5:**
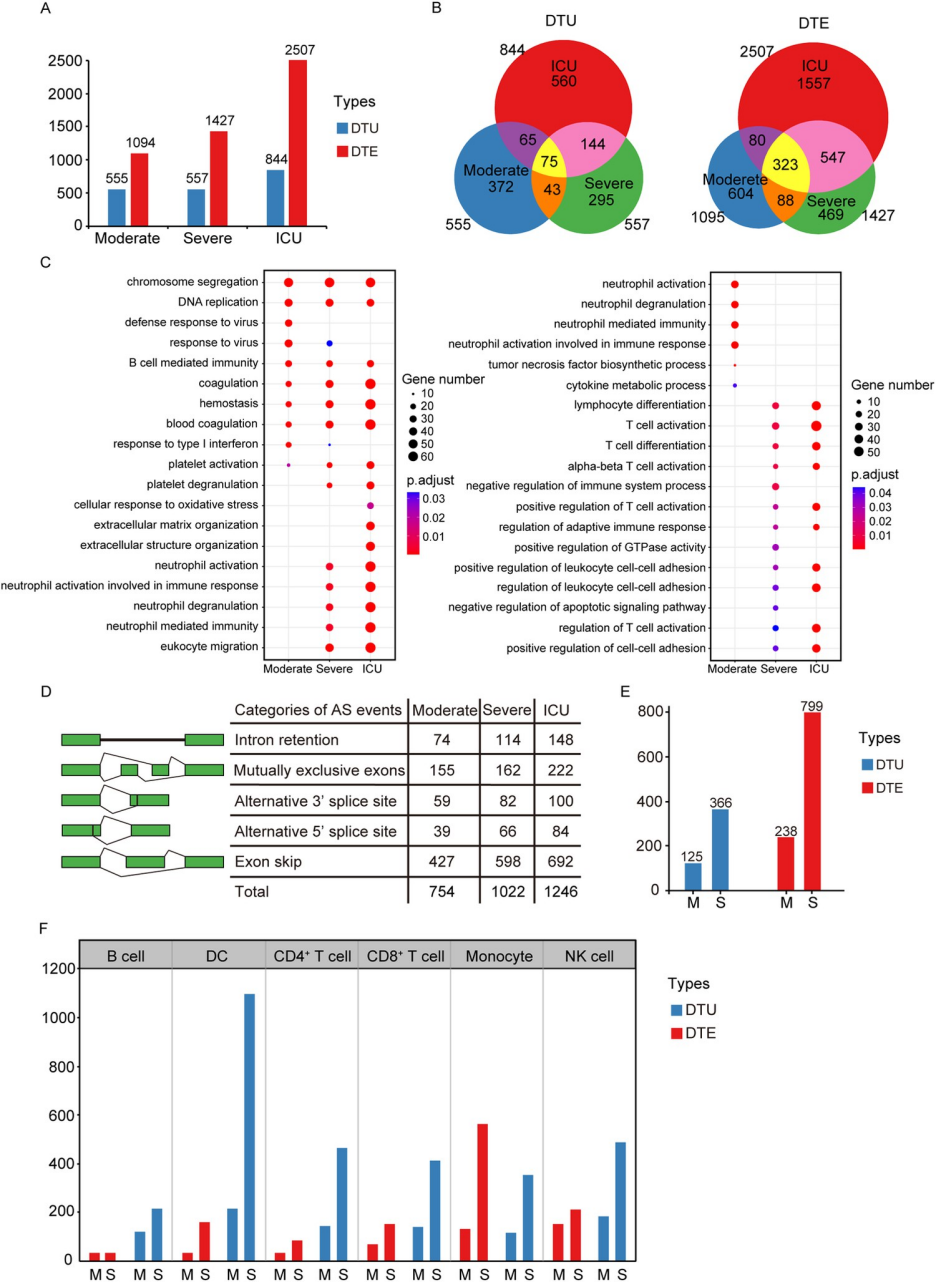
Transcriptional profiling reveals widespread dysfunction of cellular gene expression in PBMCs of COVID-19 patients. (A) Histogram diagram depicts number of DTU and DTE transcripts across three COVID-19 stages. (B) Venn diagrams depict overlap among DTU or DTE transcripts across three COVID-19 stages. (C) Dotplot visualization of the enriched GO terms of upregulated (left) and down-regulated (right) DTEs in the lungs of COVID-19 deceased patients. The color of the dots represents the p value adjusted by Benjamini–Hochberg correction for each enriched GO term identified by Fisher’s exact test using enrichGO function in R package clusterProfiler, and the size of the dot represents the number of genes enriched in the total gene set. (D) A schematic representation of 5 types of AS events and the number of genes with defined AS events in the moderate, severe and ICU COVID-19 cases compared with controls. (E) Histogram diagram depicts number of DTUs and DTEs across different COVID-19 stages in Lee et al.’s cohorts. M, Mild; S, Severe. (F) The histogram diagram depicts number of DTUs and DTEs across different COVID-19 stages in specific cell types. M, Mild; S, Severe.

To explore whether dysregulated AS landscape is of clinical relevance, we examined the association between the degree of transcript isoform dysregulation and the severity of COVID-19. We performed transcriptome analysis on the peripheral blood mononuclear cells (PBMCs) samples from a cohort of 16 patients with varying severity of COVID-19 (4 moderate, 8 severe and 4 ICU cases) and 17 healthy controls [25]. By the MMSEQ and MMDIFF analyses [38,39], we observed pervasive DTU (n = 555, 557 and 844 transcripts) and DTE (n = 1,094, 1,427 and 2,507 transcripts) changes in moderate, severe, and ICU stages compared with the controls, respectively (Fig 5A). These results revealed a continuous dysregulation of the significantly differential expression transcripts across moderate to ICU stages of COVID 19 patients. Notably, the increasing pattern of DTU and DTE numbers was also corroborated in Lee et al.’ [40] and Bernardes et al.’s [41] datasets using PBMCs obtained from COVID-19 patients with different clinical disease phases and healthy donors (Figs 5E and S9A). In Lee et al.’s single-cell RNA sequencing dataset (6 severe, 4 mild and 4 healthy donors), we also performed DTU and DTE analysis to identify immune cell types (B cell, DC, CD4^+^ T cell, CD8^+^ T cell, monocyte and NK cell) associated with the development of severe COVID-19 infection. For each of the immune cell subtypes, we compared DTU and DTE changes between disease groups and healthy donors. We observed pervasive DTU and DTE between the two sample groups (Fig 5F). More importantly, unlike the limited DTU and DTE changes shown in mild COVID-19 patients, significant changes were observed in severe COVID-19 patients across all cell types among PBMCs, including B cell, DC, CD4^+^ T cell, CD8^+^ T cell, monocyte and NK cell (Fig 5F). Examination of transcript changes in these single-cell RNA sequencing data suggested that SARS-CoV-2 mediates comprehensive dysregulation of the host immune cell transcriptions. In Bernardes et al.’s dataset, the DTU and DTE numbers were co-reached the peak in the complicated phase rather than the critical phase of COVID-19 patients (S9A Fig), suggesting that the restoration of dysregulated AS lagged behind the disease course. In a large cohort of blood samples collected from 128 patients (COVID-19: n = 51 ICU, n = 51 Non-ICU; Non-COVID-19: n = 26) with moderate to severe respiratory issues [42], we observed the elevated DTE numbers correlate with worse clinical outcomes in COVID-19 patients (S9B Fig), suggesting that COVID-19 patients represent a different transcriptomic phenotype compare to non-COVID-19 patients. In addition, at the DTU-level, the cross-stage overlap was significantly attenuated (Fig 5B), suggesting that alternative transcript usage confers a substantial portion of phase specificity of COVID-19. We further performed GO enrichment analysis of significantly up- and down-regulated DTE genes in moderate, severe and ICU compared with controls, respectively. There are only several shared pathways among these three stages, such as ‘chromosome segregation’, ‘DNA replication’, ‘B cell mediated immunity’ and four hemostasis related terms (Fig 5C).

The gene number of coagulation-related terms showed a strong correlation with the severity of COVID-19, suggesting that the disruption of coagulation signature plays a pivotal role in exacerbating the process of COVID-19. Notably, two enriched pathways in the moderate stage related to the response to virus (Fig 5C), suggesting that the host tries to establish an efficient antiviral response to prevent viral replication in the early infection phage. Interestingly, the neutrophil signature was downregulated in moderate cases but upregulated in severe and ICU cases (Fig 5C), which is consistent with our observation in the lungs of fatal COVID-19 patients. Furthermore, we used rMATS [18] to identify significant changes of AS among the three stages. In line with previous results, we observed widespread AS events across moderate, severe, and ICU (n = 754, 1,022, and 1,246 genes, respectively; Fig 5D and S4 Table), with the vast majority of these changes detected as ES events. Moreover, we observed a significant ES event of *LRRFIP1* in the ICU cohort (p = 0.009), suggesting that AS of *LRRFIP1* is a prevalent signature in severe COVID-19 patients. Altogether, virus-induced transcript isoform changes are widespread in PBMC samples of COVID-19 patients and show a strong correlation with the severity of disease process.

### AS affects drug-protein interactions

**Fig 6:**
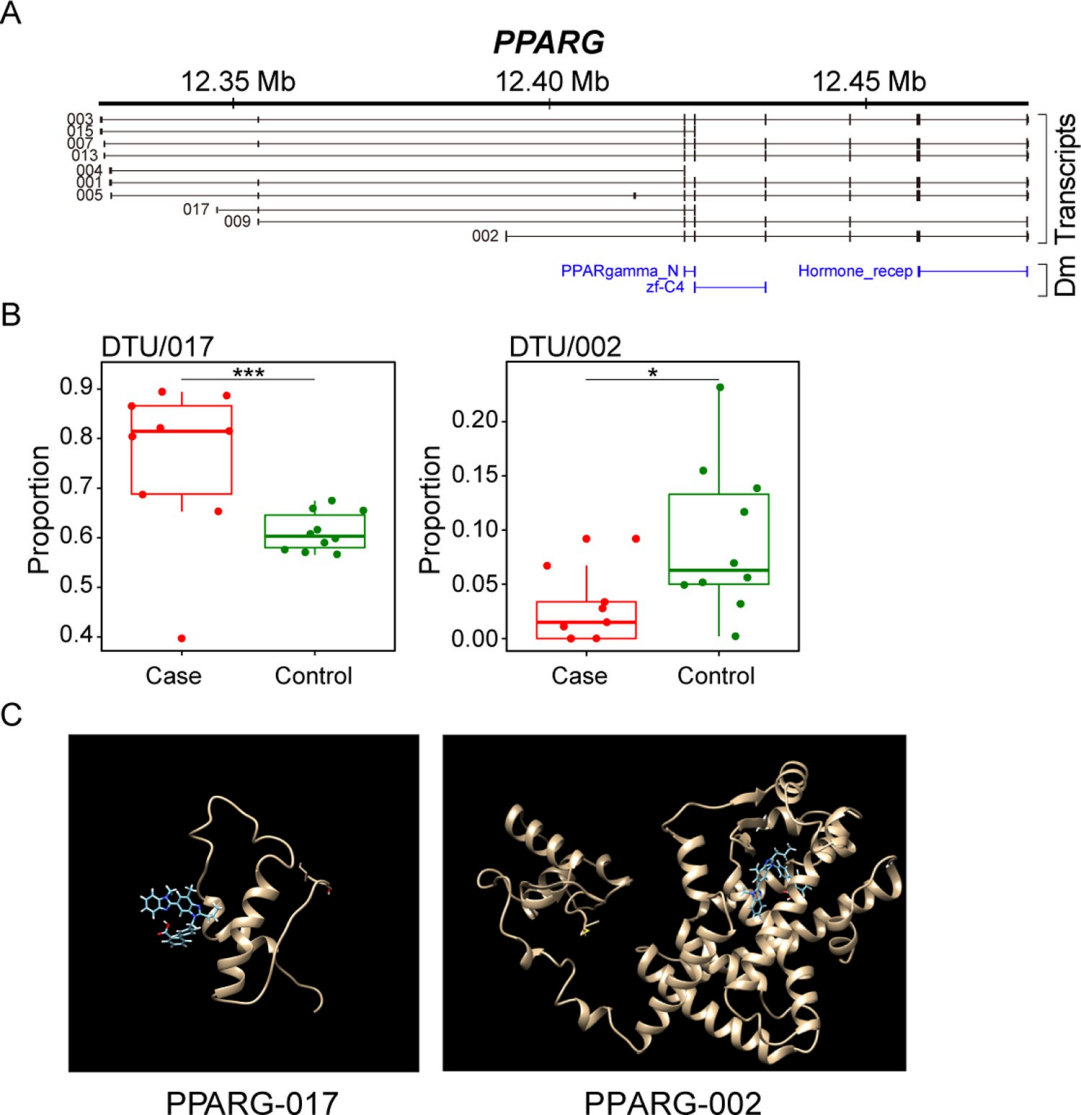
Aberrant isoform usage and drug-protein interactions of PPARG in COVID-19. (A) Transcript isoforms and domains of PPARG on human GRCh37/hg19. PPARG-002 is the canonical transcript. (B) Bar plots for changes in transcript usage for PPARG-017 and PPARG-002. * posterior probability > 0.5; ** posterior probability > 0.7; *** posterior probability > 0.9. (C) Telmisartan-protein interactions in PPARG-017 and PPARG-002.

Finally, we investigated whether AS could potentially affect the interaction between host proteins and drugs. We used SwissDock [43] to simulate the binding profile of different isoforms of drug target genes with small molecule compounds. PPARG primarily regulates lipid and glucose metabolism but also represses the inflammatory process [44]. It has been suggested that combinational use of synthetic (e.g., rosiglitazone and pioglitazone) and nutritional (e.g., eicosapentaenoic acid and carvacrol) compounds (S5 Table) that activate PPARs can inhibit the cytokine storm [45]. However, we observed significantly increased usage of isoform 017 that lacks exon 4–7 including the zf-C4 and hormone receptor domains (Fig 6A and 6B), which mediate ligand specificity and crucial for the stimulation of PPARG by drug molecule. Using structural docking analysis, we found that isoform 002 displayed a stronger interaction with telmisartan molecules than that of isoform 017 (Full fitness -2483.2876 and -521.3358, respectively; Fig 6C). These changes may result in a weak response to immunomodulatory therapies in the severe COVID-19 patients. Moreover, the canonical isoform 002, which displays higher usage in normal tissues than in the COVID-19 samples (Fig 6B) and has complete hormone receptor domain (Fig 6A), may function as a regulator or suppressor on the cytokine storm under virus infections.

## Discussion

The present study demonstrated that SARS-CoV-2 infection extensively modifies the splicing patterns of numerous cellular transcripts. These splice variants altered their relative abundance of different isoforms and encoded different proteins that are critical for host defense. These data open new avenues of research for a better understanding of post-transcriptional events during SARS-CoV-2 infection and possible new targets for the development of antiviral agents.

Recent studies have shown that cellular spliceosome is a frequent viral target [13,26,46]. snRNPs U1-U6 constitute the core components of the spliceosome, and SR and hnRNPs are regulators of constitutive and alternative splicing. Our results showed that six spliceosomal component proteins (DDX5, DDX17, DDX39B, PRPF6, SNRNP40, and SNRNP200) were significantly down-regulated in COVID-19 patients. Previous studies have noted that NS5 from Dengue virus interacts with DDX23 from the U5 snRNP particle to modulate the host splicing machinery [7]. Similarly, Arora et al. reported that SARS-CoV-2 hijacks the DDX58 that is involved in miRNA biogenesis and mRNA splicing to promote its replication [6]. Our study did not identify DDX23 and DDX58, but did identify DDX5, DDX17, and DDX39B. These results suggest that SARS-CoV-2 can hijack the host spliceosome components to modulate cellular AS in the lungs of COVID-19 patients, which is corroborated by Banerjee et al.’s observation that SARS-CoV-2 protein NSP16 binds mRNA recognition domains of U1/U2 snRNAs and disrupts mRNA splicing [26]. In addition, several viruses modulate host splicing factors to enhancing viral replication. For example, hnRNP C1/C2 during Dengue virus infection [47], hnRNP K during Dengue/Junìn virus infections [48], hnRNP D during West Nile virus infection [49], and hnRNP M during picornaviruses infections [50] all acts as positive regulators of viral replication. We identified that 13 hnRNP proteins (HNRNP A0, A2B1, A3, DL, F, H1, H2, L, LL, R, U, UL1, and UL2) were down-regulated in our lung cases, suggesting that host splicing factors may act as a role to restrict viral replication in these fatal COVID-19 patients. These results are consistent with our previous observation that these case samples had a very low viral load and patient deaths may be more related to an overexuberant and uncontrolled host immune response rather than unabated viral replication [28].

Viruses modulate the host splicing machinery that can cause a widespread dysfunction of host cell expression. We found that a large number of DTUs and DTEs related to blood coagulation, neutrophil activation, response to oxidative stress and cytokine production in the lungs of fatal COVID-19 patients. Recent studies have reported that the clinical characteristics of severe COVID-19 patients are coagulation abnormalities, neutrophilic infiltrate, acute respiratory distress syndrome (ARDS) and cytokine storm, suggesting that the alternation of transcript is strongly correlated with the clinical manifestations [28,51,52]. In addition, isoform switch was predicted to result in functionally different RNAs/proteins, such as IL2. Understanding the isoform regulation may help inform future clinical trials for COVID-19 therapeutics. For example, our finding that the canonical transcript usage of IL6 is not increased in the lungs of these fatal cases highlights that anti-IL-6 therapies may not be an effective treatment for severe, late-stage COVID-19 pneumonia, which is consistent with preliminary randomized clinical trial findings [53,54]. Moreover, different isoforms of PPARG exhibit distinct drug-binding sites, suggesting that targeting each individual protein isoform might represent an efficient splicing isoform-specific therapy. Notably, most clinical trials for COVID-19 focus on one or several proteins ignoring the multiple splice-variant or protein-isoforms, which might contribute to unexpected therapeutic activity or adverse side effects.

The most commonly predicted consequences of AS were large changes in amino acid sequence (corresponding to loss or gain of amino acid coding exons) and change of protein domains. Importantly, the distribution of protein domain gain versus domain loss was skewed toward domain loss for key immune regulated genes in severe COVID-19 patients. For example, an exon skipping event in COVID-19 disrupts a thyroglobulin domain in *CD74*. The *CD74* thyroglobulin domain inhibits cathepsins [55], whereas cathepsin and serine proteases are critical for the entry of coronaviruses, including SARS-CoV-2 [56], suggesting that this may be the mechanism for antiviral activity by inhibiting viral entry. Similarly, the ES event disrupts a domain in *LRRFIP1*, changes that may affect the ability to mediate the production of type I interferon [57]. These results highlight that during global regulation of AS upon infection, targets do not get randomly selected for altered splicing; instead, they are specially chosen, which enhances the chances of pathogen survival. Altogether, these results suggest that the exon-specific detection of AS might serve as a reliable biomarker and provide a novel approach to diagnose and monitor COVID-19 progression.

## Materials and methods

### Ethics statement

The study protocol was approved by the Tongji Hospital affiliated to Tongji Medical College of Huazhong University of Science and Technology Institutional Review Board (approval TJ-IRB20200341).

### Human lung samples

Eight patients who died at Jinyintan Hospital (Wuhan, China) and one patient who died at Central Hospital of Wuhan as a result of COVID-19 underwent an autopsy, during which samples were taken from representative areas of the lung for routine histologic analysis. The control (uninfected) lung (n = 10) represent healthy tissue obtained from patients with lung cancer during biopsy and/or surgical resection performed as part of routine clinical care. These control patients were matched to the cases with respect to age, sex, and past medical history. Lung tissues were fixed with 10% formalin for 3 days at room temperature, trimmed into appropriate size and shape, and embedded in paraffin.

### Protein extraction and digestion

Protein extraction from formalin-fixed paraffin-embedded (FFPE) tissues was performed according to the previous methods [58,59]. Briefly, samples were dewaxed in xylene and pelleted by centrifugation. Pellets were resuspended in lysis buffer (1% sodium dodecyl sulfate, 200 mM Tris·HCl pH 8, 1% protease inhibitor mixture). After sonication for 5 min (3 s on, and 5 s off) at 30% amplitude, the tubes were heated at 100°C for 30 min and subsequently centrifuged for 10 min at 20,000x*g*. The supernatant containing the extracted proteins was recovered and quantified using a bicinchoninic acid test. Proteins were reduced in 5 mM dithiothreitol at 56°C for 30 min and alkylated at room temperature in 11 mM iodoacetamide for 15 min in the dark. Then the proteins were added to the filter membrane for filter-aided sample preparation. The membrane was washed 3 times with 8 M urea and then washed 3 times with 20 mM NH_4_HCO_3_ at 12,000 x*g*. A total of 100 μL of 20 mM NH_4_HCO_3_ containing trypsin (protein:trypsin = 50:1, m/m) was added to the membrane and incubated overnight at 37°C. Peptides were harvested from the membrane by centrifuging for 10 min at 12,000 x*g* and then desalted using C18 precolumn and finally dried down using a vacuum centrifuge. All samples were stored at -80°C for further analysis.

### LC-MS/MS assay

The tryptic peptides were dissolved in solvent A (0.1% formic acid in water), directly loaded onto a home-made reversed-phase analytical column (25-cm length, 75 μm internal diameter). Peptides were separated with a gradient from 4 to 6% solvent B (0.1% formic acid in acetonitrile) in 2 min, 6 to 24% over 68 min, 24 to 32% in 14 min, and climbing to 80% in 3 min, then holding at 80% for the last 3 min, all at a constant flow rate of 300 nL/min on a nanoElute high-performance liquid chromatography system (Bruker Daltonics).

The peptides were subjected to capillary source followed by the timsTOF Pro (Bruker Daltonics) mass spectrometry. The electrospray voltage applied was 1.60 kV. Precursors and fragments were analyzed at the TOF detector, with a MS/MS scan range from 100 to 1,700 m/z. The timsTOF Pro was operated in parallel accumulation serial fragmentation (PASEF) mode. Precursors with charge states 0 to 5 were selected for fragmentation, and 10 PASEF-MS/MS scans were acquired per cycle. The dynamic exclusion was set to 30 s.

### Proteomics database search

The resulting MS/MS data were processed using Maxquant search engine (v.1.6.6.0). Tandem mass spectra were searched against the Uniprot human database (20,366 sequences, released at 2020/05) concatenated with reverse decoy database. Trypsin/P was specified as cleavage enzyme allowing up to 2 missing cleavages. The mass tolerance for precursor ions was set as 40 ppm in both first search and main search, and the mass tolerance for fragment ions was set as 0.04 Da. Carbamidomethyl on Cys was specified as fixed modification, and acetylation on protein N-terminal and oxidation on Met were specified as variable modifications. Peptide and protein identified with False Discovery Rate (FDR) < 1%.

### Analysis of proteomic data

Proteins that were differentially expressed between cases and controls were identified with Student’s *t* test p value < 0.05 and |log_2_FC| > 1. Volcano plots were generated using the R package ggplot2. To assess interaction of splicing-related genes, we interrogated the list of 244 cellular spliceosomal components defined in a study [60]. The interaction network between cellular spliceosomal components and viral proteins was built with Cytoscape v3.7.2 [61].

### Library preparation and sequencing

Total RNA was isolated from FFPE lung tissue from cases and controls using the RNeasy FFPE kit (Qiagen) according to the manufacturer’s instructions with a minor modification. Thin tissue sections weighing ~0.1 mg were used for each sample, and an additional genomic DNA removal step was performed by treating the sample with 2 units of TURBO DNA-free DNase (Invitrogen) for 30 min at 37°C. The Trio RNA-Seq Library Preparation Kit (Nugen; Redwood City, CA) was used to generate sequencing libraries as per the manufacturer’s instructions, with 1 to 5 ng RNA used as input. The library size and quality were assessed using an Agilent Bioanalyzer 2100. Pooled libraries were sequenced on an Illumina NextSEq 550 using a 75-cycle kit with single-end read mode.

### Analysis of RNA-seq data

Raw reads were trimmed using Trim Galore (v0.6.4, https://github.com/FelixKrueger/TrimGalore) with parameters “quality 20, stringency 3, length 20” to remove both poor-quality calls and adapters. Quality-controlled reads were mapped to the transcriptome with Kallisto v0.46.2 [62], using Ensembl v70 as a reference. Following the estimation of transcript expression levels with MMSEQ v1.0.9 [38], its companion tool MMDIFF [39] was used to identify both DTU and DTE. MMDIFF uses Bayesian inference to evaluate the probability that two transcripts are differentially used across conditions, which is termed ‘posterior probability’. Transcripts with posterior probability > 0.5 (calculated by MMDIFF), |log_2_(fold change)| > 1 and counts per million mapped reads (CPM) > 1 in at least three of the samples were considered differentially expressed. Transcripts with posterior probability > 0.5 and |proportional change| > 0.1 were considered differentially used. For the single-cell RNA sequencing data, the 6 immune cell type reads were exactly extracted from the raw fastq files based on cell barcode sequences. All box and whisker plots were generated using the R package ggplot2. The box depicts the first to the third quartile, and the horizontal line indicates the median. Interquartile range (IQR) was calculated as the third quartile subtracted by the first quartile. The whiskers extend to the most extreme dataset value that is within 1.5 times the IQR of the box. The data points outside the whiskers represents outliers.

We used Leafcutter v0.2.9 [17] to leverage information from reads that span introns to quantify clusters of variably spliced introns. From the already aligned FASTQ files by STAR, output bam files were converted into junction files. Intron clustering was performed using default settings of 50 reads per cluster and a maximum intron length of 500 kb. P-values were corrected for multiple testing using the Benjamini-Hochberg (BH) method and used to select clusters with significant splicing differences (P < 0.05). Schematic visualization of significant intron clusters was done using the leafviz R shiny package (https://davidaknowles.github.io/leafcutter/articles/Visualization.html). Analysis of event-level differential splicing was performed using rMATS v4.0.2 [18]. PSI values imply reduced inclusion of alternative exons and denote the percentage of proteins that contain the exon compared to the total transcript population. The PSI is calculated using:

$$\mathrm{PSI}=\frac{\mathrm{IR}}{\mathrm{IR}+\mathrm{ER}}$$

Where IR is inclusion reads and ER is exclusion reads. PSI values were calculated for several classes of AS events, including skipped exon (SE), alternative 5′ splice site (A5SS), alternative 3′ splice site (A3SS), mutually exclusive exons (MXE), and retained introns (RI). P-values < 0.05 indicated significant AS events. Significant AS events with PSI > 0 in at least 50% of the samples were used to construct co-splicing network. AS events with Pearson correlation coefficient > 0.5 and P < 0.01 were input for module detection with MONET [36]. These modules were visualized as a co-splicing network in Cytoscape v3.7.2 [61]. Histogram plots were generated using the R package ggplot2. Venn diagrams area-proportional to the number of genes with significant DTE and DTU in each group were then created using the eulerr R package.

In order to have a better understanding of the interactions between the protein isoforms and ligands (drugs), we constructed 3D structures of different protein isoforms by their primary sequences from Ensembl v70 and using SWISS-MODEL [63] built homology models. Then use 3Drefine (http://sysbio.rnet.missouri.edu/3Drefine/) to refine the homology models and get the structure of target drugs from DrugBank database (https://go.drugbank.com/). Finally, SwissDock [43] was used to find the ligand-protein binding mode. The full fitness scores for SwissDock poses were investigated, with lower scoring poses being more favorable than higher scoring poses [64].

### CIBERSIRTx estimation

CIBERSORTx online analysis platform (https://cibersortx.stanford.edu/) was applied to infer cell-type-specific gene expression profiles without physical cell isolation [29]. To construct a custom signature matrix, we prepared and uploaded the single-cell expression matrix of lethal COVID-19 (accession number GSE171524) according to the instructions with CIBERSORTx. The default parameters remained. Then we ran “CIBERSORTx” and obtained a signature matrix of 9 cell types from scRNA-seq data. Next, we will use the signature matrix we create to infer cell fractions from our bulk RNA-seq samples. We set permutations to 1,000. Other parameters retained the default. After CIBERSORTx, the relative proportions of 9 subsets of cell types in each sample was downloaded and plotted with R programming language.

### Functional enrichment analysis

Pathway analysis of the differentially expressed proteins and transcripts was performed using the R package clusterProfiler [65], with org.Hs.eg.db used for annotation. Enriched gene ontology (GO) pathways were identified as GO terms that had a P-adjusted-value < 0.05 after BH correction for multiple testing. Biological interpretations of DTUs were carried out using the GO public database with the use of biological process (BP) category using ViSEAGO R package [66]. Enrichment tests were performed using Fisher test, subsequently with classic, elim, weight and weight01 algorithms. Enriched GO terms (P < 0.01) were grouped into functional clusters using hierarchical clustering based on Wang’s semantic similarity between GO terms respecting GO graph topology and Ward’s criterion. To go further in the interpretation, these functional clusters were grouped using hierarchical clustering based on BMA distance between sets of GO terms and Ward’s criterion.

### Splicing and DTE validation

For AS analysis, selected exon-skipping events were validated by semiquantitative RT–PCR in 9 COVID-19 and 10 control samples. Total RNA (1–2 μg) was treated with 1 unit of Baseline-ZERO DNase (Lucigen), cleaned up with 1.8x AMPure XP (Beckman Coulter), and reverse-transcribed using SuperScript III reverse transcriptase and random hexamer primers (Invitrogen). After clean-up with 1.8x AMPure XP, AS events were PCR amplified from 20 ng of cDNA for 30 cycles in 25 μl volume containing exon-specific primers at a concentration 0.5 μM each, and ChoiceTaq Blue MasterMix (Denville) according to manufacturer instructions. Exon-specific PCR primers (S6 Table) were designed in the flanking exons of each skipping event using Primer3 [67] and BLAST [68]. PCR products were cleaned up with 1.8x AMPure XP (Beckman Coulter) and analyzed on DNA 1000 chips on an Agilent 2100 Bioanalyzer system. Peaks corresponding to the amplicon including or excluding the skipped exon were quantified using the Bioanalyzer Expert software, and PSI ratios were calculated by dividing the molarity of the lower band (exon skipped) by the sum of the molarity of the lower and upper band (exon included). The dPSI between cases and control for each event was calculated as the difference between the average PSI in cases and average PSI in controls. Sample details and primers are reported in S6 Table.

For DTE analysis, selected transcripts were validated by semiquantitative RT-PCR using a similar approach as for AS. The molarity of the transcript bands was used for the DTE analysis between case and control samples. Sample details and primers are reported in S6 Table.

## Supporting information

S1 Fig Interaction network between cellular spliceosomal components and viral proteins. Nodes and edges between nodes represent protein and protein–protein interactions, respectively. Light blue and orange nodes represent core or regulatory spliceosomal proteins, and other nodes represent viral proteins. The 13 spliceosomal components that are differentially expressed between cases and controls are named.
(TIF)

S2 Fig Transcript isoform usage of blood coagulation genes between cases and controls. (TIF)

S3 Fig Transcript isoform usage of RNA splicing genes between cases and controls. (TIF)

S4 Fig Transcript isoform usage of immunity genes between cases and controls. (TIF)

S5 Fig **Dotplot visualization of enriched top 30 GO terms of up- (A) and down-regulated (B) DTEs in cases.** The color of the dots represents the p-value adjusted by Benjamini-Hochberg for each enriched GO term identified by Fisher’s exact test using enrichGO function in R package clusterProfiler, and the size of the dot represents the number of genes enriched in the total gene set.
(TIF)

S6 Fig Representative Agilent 2100 Bioanalyzer gel images (DNA 1000 chips) obtained for splicing validation. (TIF)

S7 Fig Validation of differential transcript expression. (A) Comparison of molarity obtained from semiquantitative PCR results for 4 transcripts tested in 9 COVID-19 and 10 control samples. * P < 0.05; ** P < 0.01; *** P < 0.001. (B) Representative Agilent 2100 Bioanalyzer gel images (DNA 1000 chips) obtained for DTE validation.
(TIF)

S8 Fig Gene co-splicing networks capture shared and disease-specific cellular processes and interactions. (A) Neutrophil activation enriched in case and control module separately, with the hub gene *DDX3X* shown. Orange and blue nodes represent case or control specific AS genes and green nodes represent overlapped AS genes. Edges represent co-splicing (Pearson correlation > 0.5) interactions. Nodes represent genes with significant AS events (P < 0.05). (B and C) Macrophage activation and B cell homeostasis module dysregulated between cases and controls.
(TIF)

S9 Fig **Histogram diagram depicts number of DTUs and DTEs across different COVID-19 stages in Bernardes et al.’s [41] (A) and Overmyer et al.’s [42] (B) cohorts.** Pseudotime: 1, Incremental; 2, Critical; 3, Complicated; 4, Complicated; 5, Moderate/early convalescence; 6, Late convalescence; 7, Long-term follow-up.
(TIF)

S1 Table Summary of identified proteins in COVID-19 cases and controls. (XLSX)

S2 Table Detailed of GO terms in the genes with DTU. (XLSX)

S3 Table Summary of all AS events in the lungs of fatal COVID-19 patients. (XLSX)

S4 Table Summary of all AS events in the moderate, severe and ICU COVID-19 patients. (XLSX)

S5 Table Summary of target-drugs associated with COVID-19 in DrugBank database. (XLSX)

S6 Table Splicing and DTE validation primers. (XLSX)
