# Supplementary figures and images for: Abnormal global alternative RNA splicing in COVID-19 patients
Source: PLoS Genet. 2022 Apr 14;18(4):e1010137. doi: 10.1371/journal.pgen.1010137 (PMC9089920; doi:10.1371/journal.pgen.1010137)

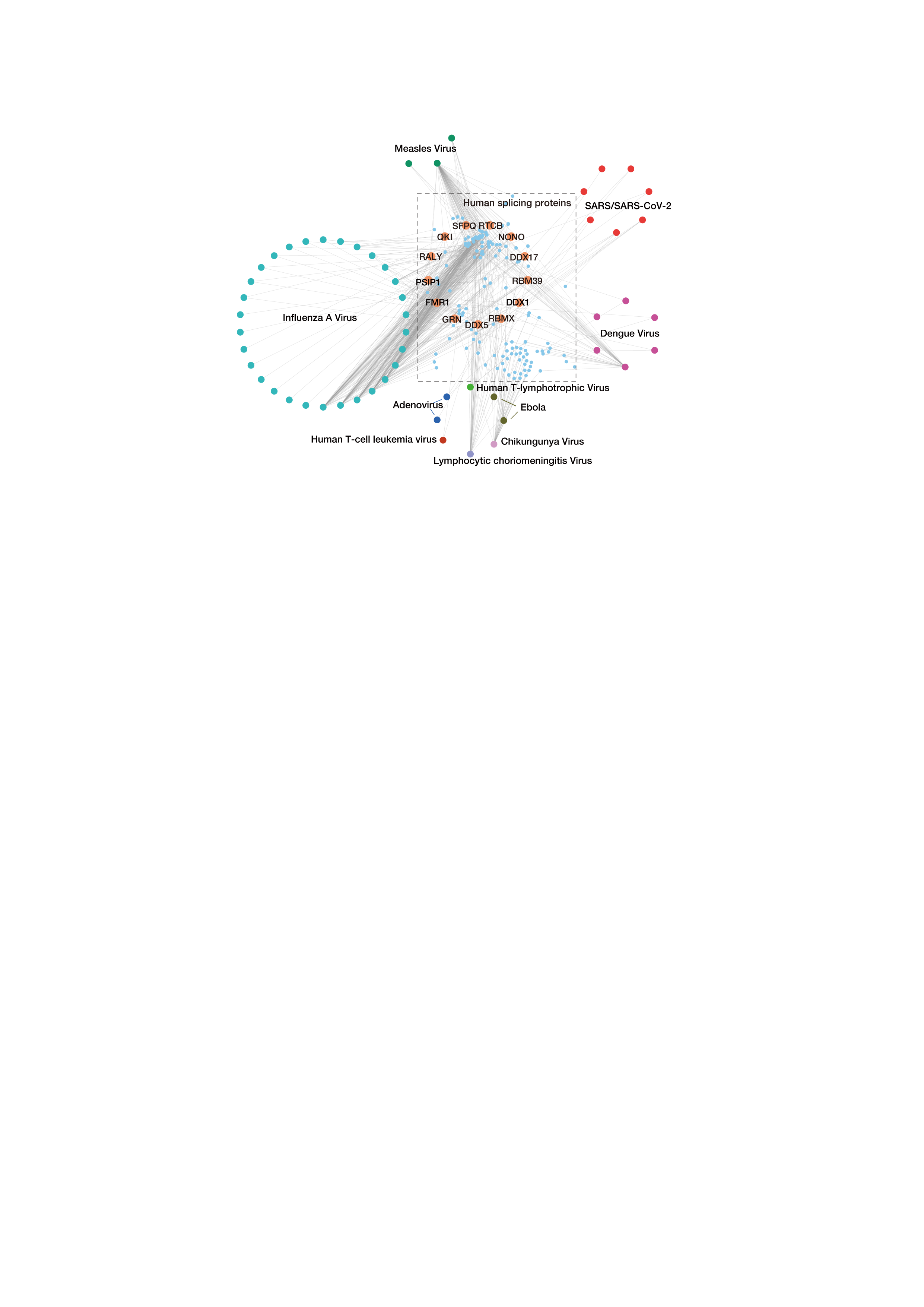

Supplement: S1 Fig — Nodes and edges between nodes represent protein and protein–protein interactions, respectively. Light blue and orange nodes represent core or regulatory spliceosomal proteins, and other nodes represent viral proteins. The 13 spliceosomal components that are differentially expressed between cases and controls are named. (TIF) [file pgen.1010137.s001.tif]

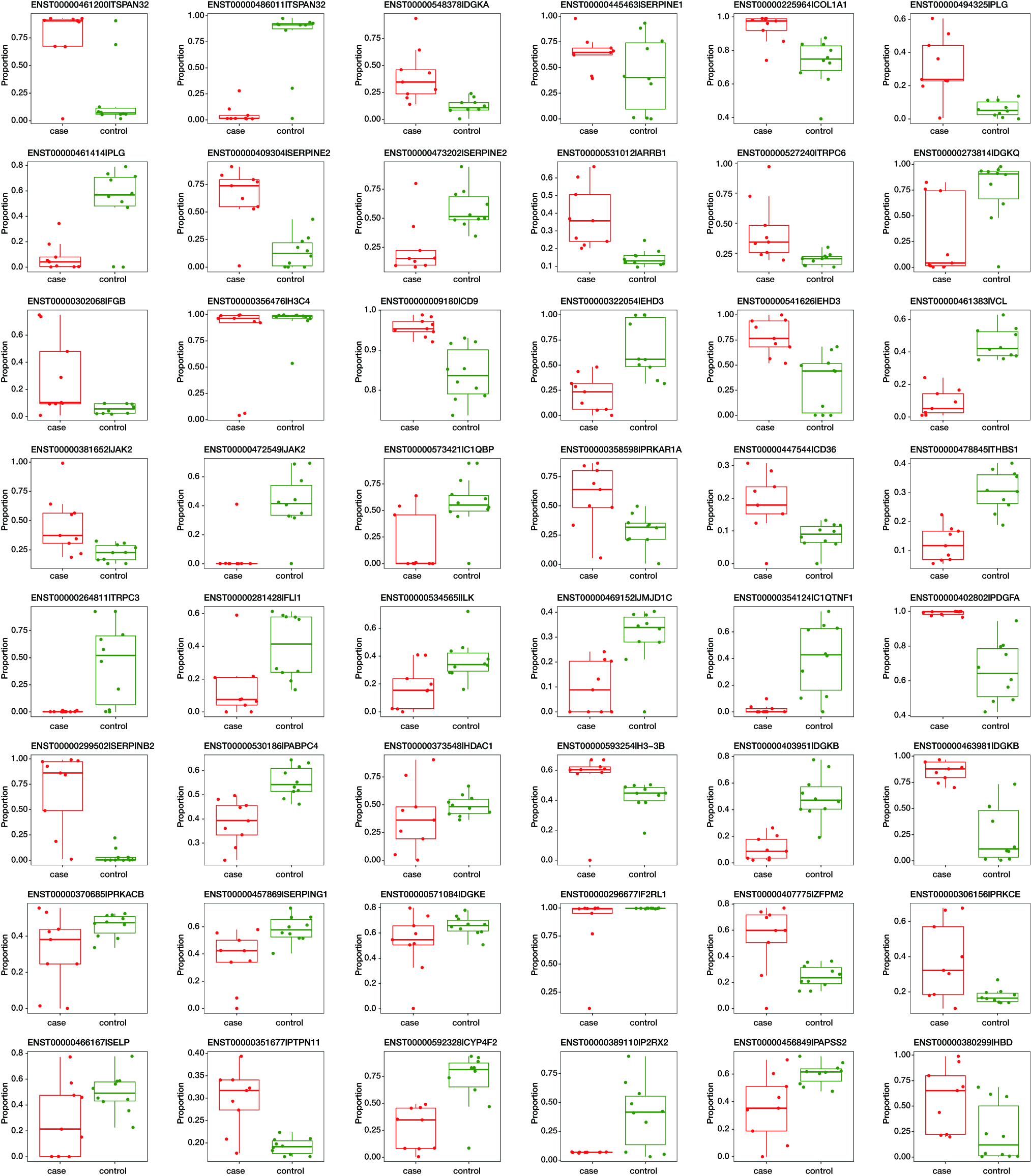

Supplement: S2 Fig — (TIF) [file pgen.1010137.s002.tif]

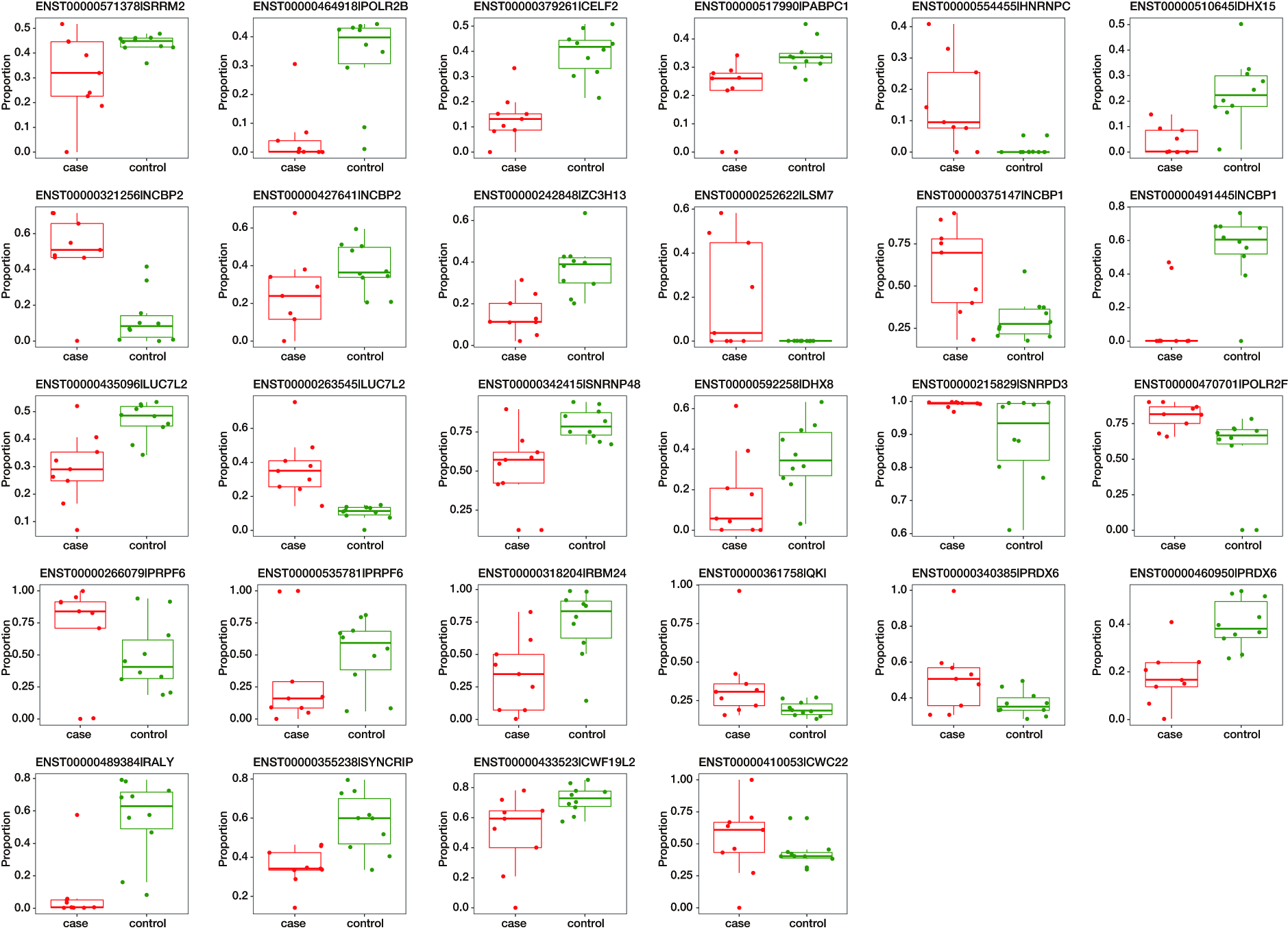

Supplement: S3 Fig — (TIF) [file pgen.1010137.s003.tif]

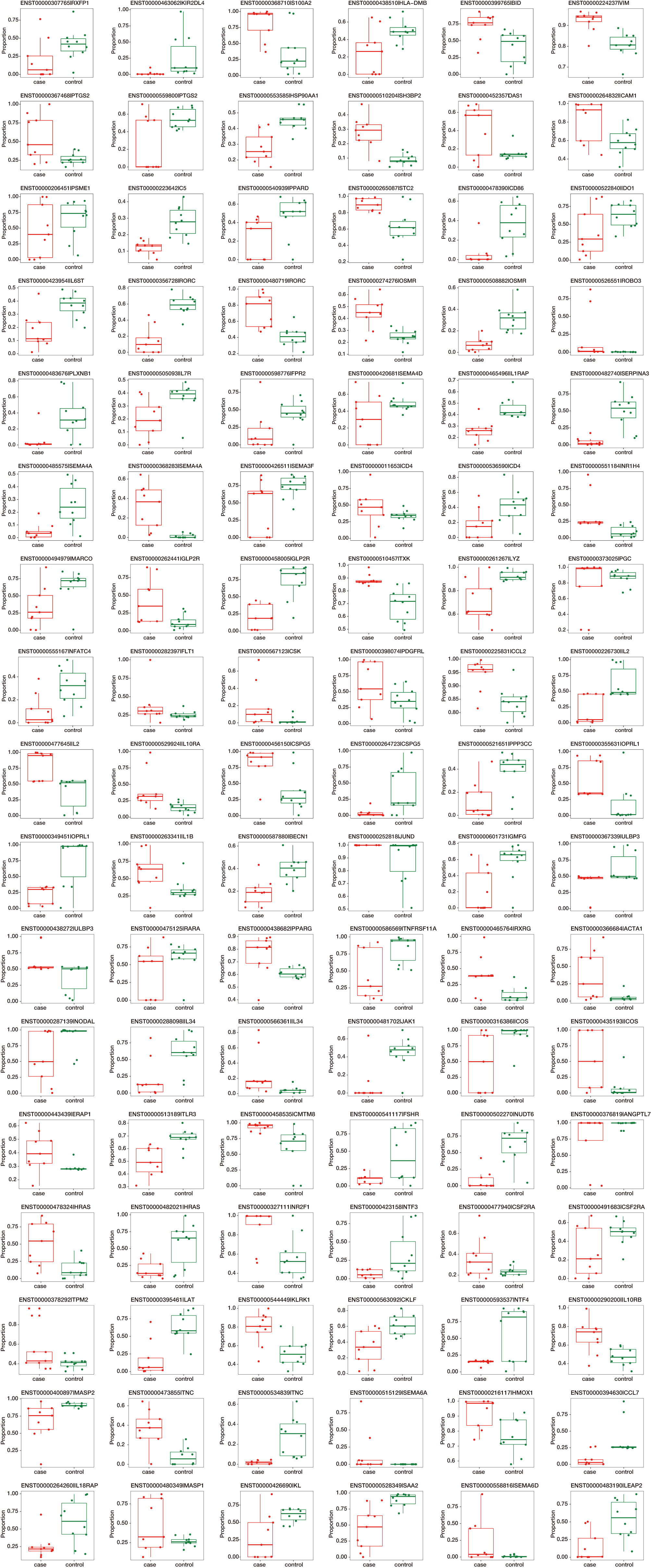

Supplement: S4 Fig — (TIF) [file pgen.1010137.s004.tif]

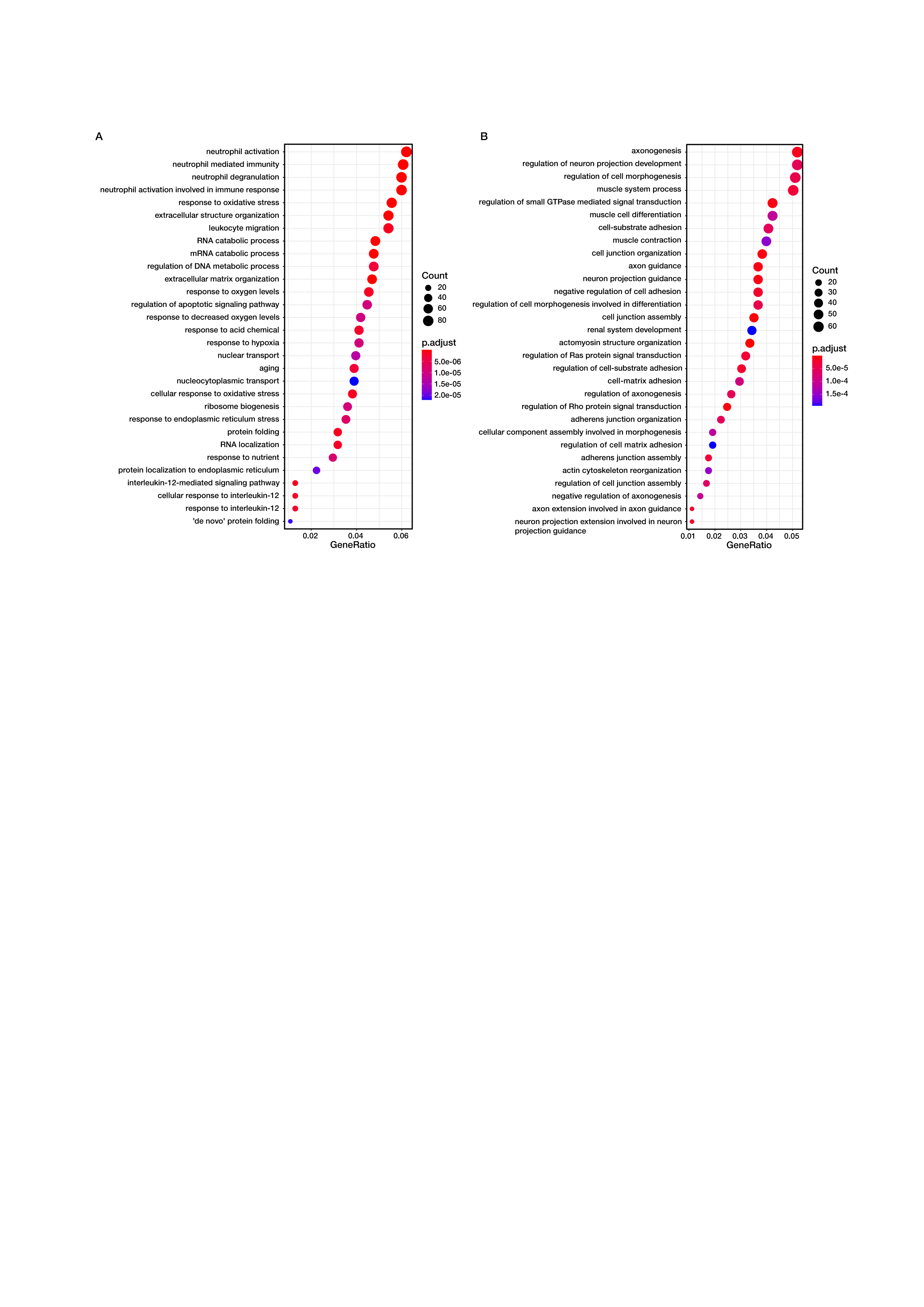

Supplement: S5 Fig — Dotplot visualization of enriched top 30 GO terms of up- (A) and down-regulated (B) DTEs in cases. The color of the dots represents the p-value adjusted by Benjamini-Hochberg for each enriched GO term identified by Fisher’s exact test using enrichGO function in R package clusterProfiler, and the size of the dot represents the number of genes enriched in the total gene set. (TIF) [file pgen.1010137.s005.tif]

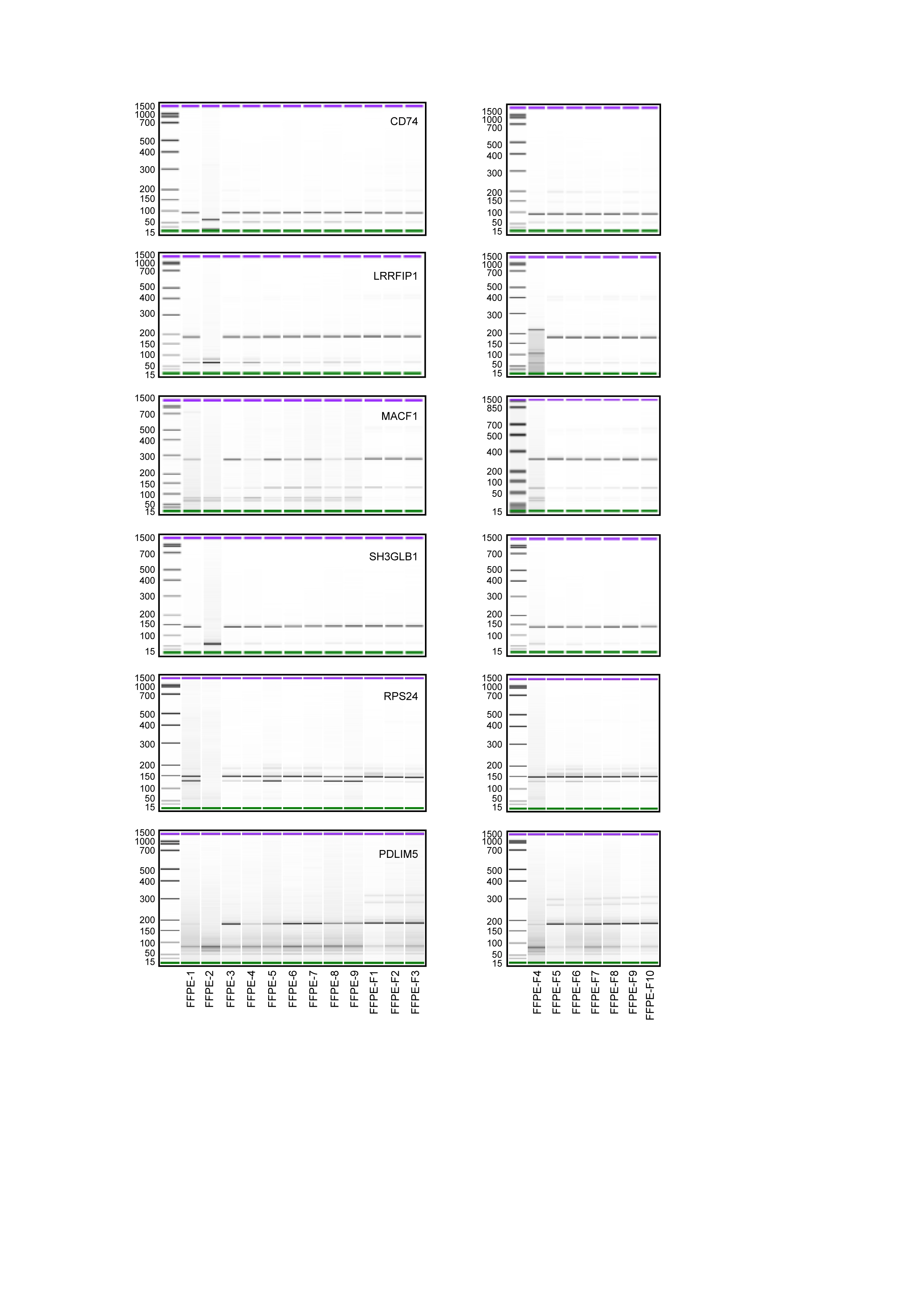

Supplement: S6 Fig — (TIF) [file pgen.1010137.s006.tif]

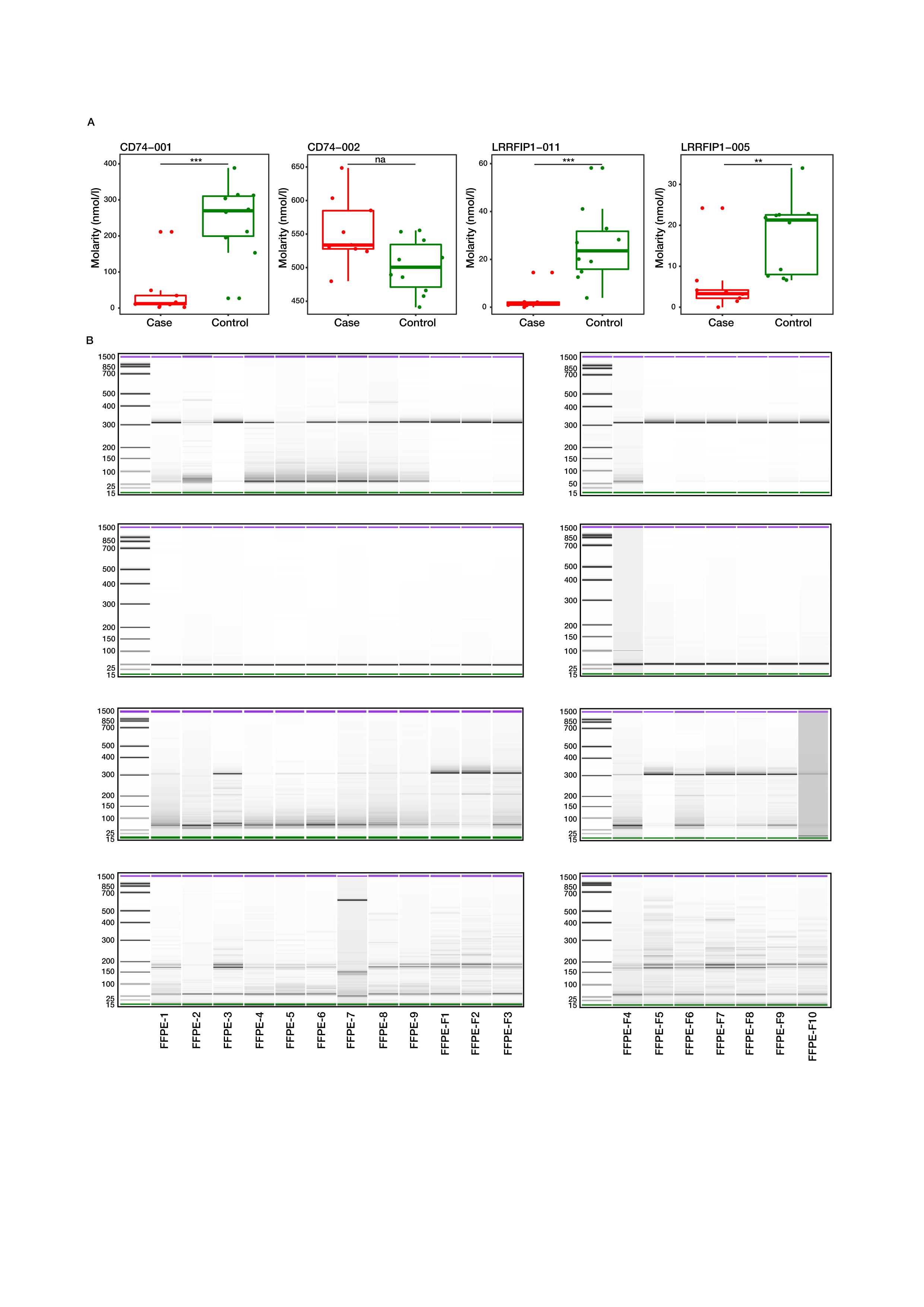

Supplement: S7 Fig — (A) Comparison of molarity obtained from semiquantitative PCR results for 4 transcripts tested in 9 COVID-19 and 10 control samples. * P < 0.05; ** P < 0.01; *** P < 0.001. (B) Representative Agilent 2100 Bioanalyzer gel images (DNA 1000 chips) obtained for DTE validation. (TIF) [file pgen.1010137.s007.tif]

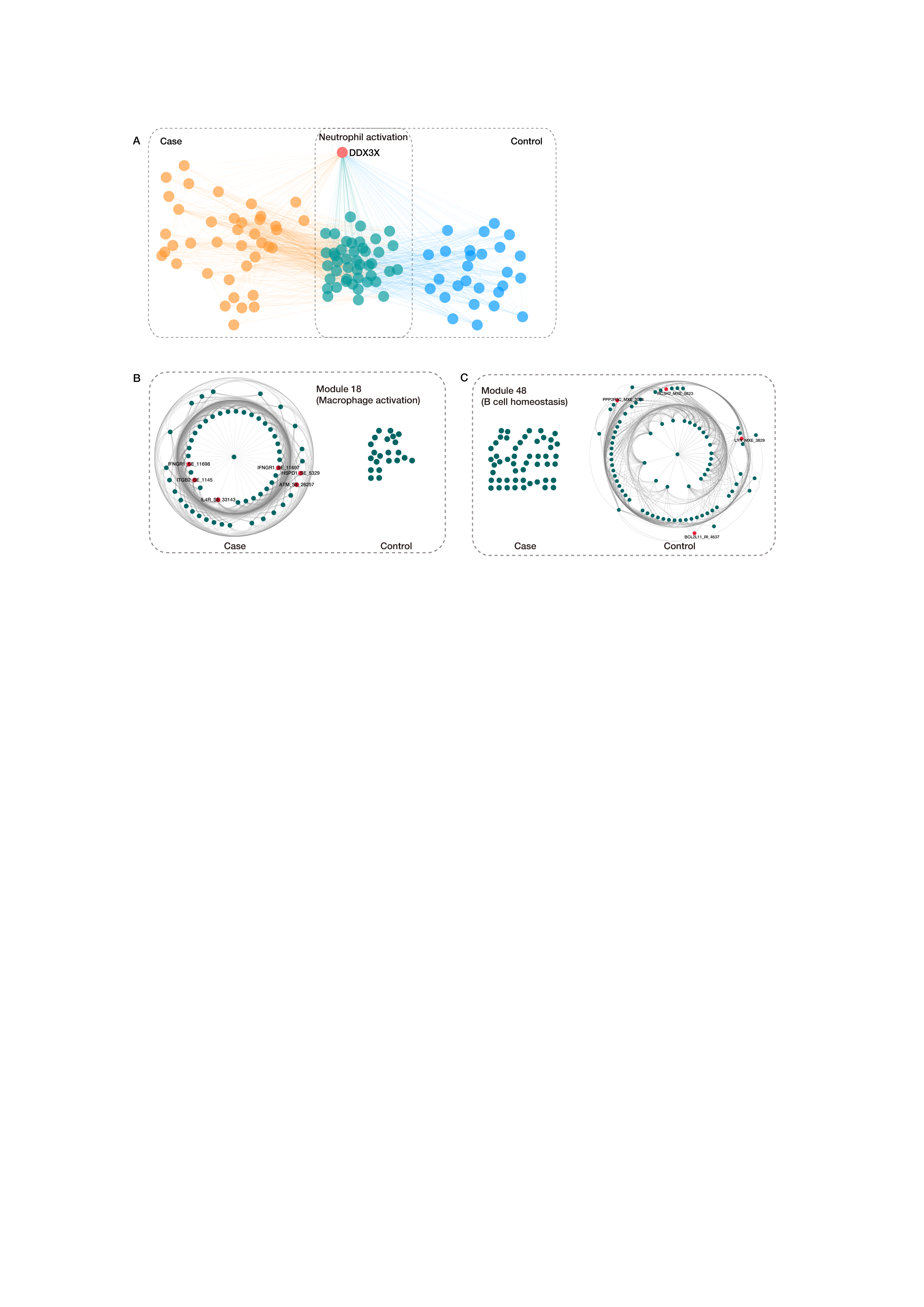

Supplement: S8 Fig — (A) Neutrophil activation enriched in case and control module separately, with the hub gene DDX3X shown. Orange and blue nodes represent case or control specific AS genes and green nodes represent overlapped AS genes. Edges represent co-splicing (Pearson correlation > 0.5) interactions. Nodes represent genes with significant AS events (P < 0.05). (B and C) Macrophage activation and B cell homeostasis module dysregulated between cases and controls. (TIF) [file pgen.1010137.s008.tif]

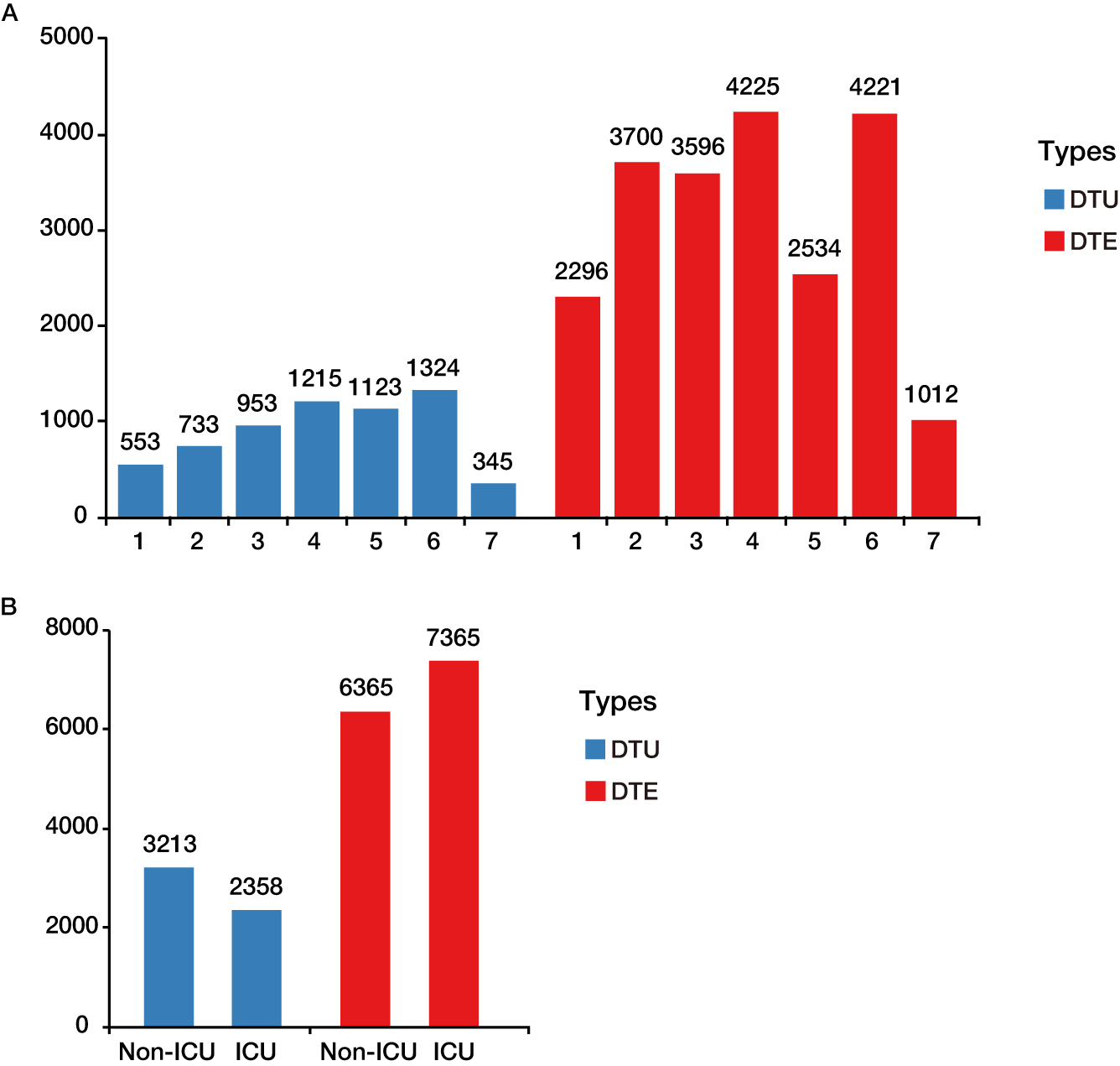

Supplement: S9 Fig — Histogram diagram depicts number of DTUs and DTEs across different COVID-19 stages in Bernardes et al.’s [41] (A) and Overmyer et al.’s [42] (B) cohorts. Pseudotime: 1, Incremental; 2, Critical; 3, Complicated; 4, Complicated; 5, Moderate/early convalescence; 6, Late convalescence; 7, Long-term follow-up. (TIF) [file pgen.1010137.s009.tif]
